# Recurarization after sugammadex reversal in a patient with amyotrophic lateral sclerosis: Case report

**DOI:** 10.1097/MD.0000000000046928

**Published:** 2026-01-02

**Authors:** Yun-Wen Wang, Ying Zhang, Xiao Hu, Xue Li, Lin Han, Dong-Xin Wang, Ting Ding

**Affiliations:** a Department of Anaesthesiology, Peking University First Hospital, Beijing, China.

**Keywords:** amyotrophic lateral sclerosis, general anesthesia, recurrent neuromuscular block, rocuronium, sugammadex

## Abstract

**Rationale:**

Amyotrophic lateral sclerosis (ALS) confers heightened and unpredictable sensitivity to nondepolarizing neuromuscular blocking agents and a high risk of postoperative respiratory failure. Although sugammadex reliably reverses rocuronium, recurarization may occur and is likely under-recognized in ALS. We report 2 ALS patients undergoing percutaneous endoscopic gastrostomy, one of whom developed delayed recurarization after apparent reversal.

**Patient concerns:**

Both women (67 and 68 years) presented with progressive dysphagia requiring percutaneous endoscopic gastrostomy. Case 1 had dyspnea, dysarthria, and long-standing noninvasive positive-pressure ventilation; Case 2 had bulbar signs without preoperative ventilatory support. The key perioperative concern in both cases was ventilatory failure from residual neuromuscular block.

**Diagnoses:**

ALS had been established clinically. In Case 2, recurarization was diagnosed shortly after extubation when acute hypercapnic respiratory failure and clinical weakness followed an earlier recovery to a train-of-four (TOF) ratio of 92%.

**Interventions:**

Intravenous anesthesia with propofol and remifentanil was used. Case 1 received rocuronium 10 mg (0.2 mg/kg) and was reversed with sugammadex 90 mg (2 mg/kg) at TOF count 0, achieving a TOF ratio of 98% within 3 minutes before extubation and postoperative noninvasive ventilation. Case 2 received rocuronium 30 mg (0.6 mg/kg) and sugammadex 200 mg (3.8 mg/kg) at TOF count 1, recovered to a TOF ratio of 92% at 4 minutes, but developed respiratory failure 3 minutes after extubation; mask ventilation and neostigmine 2 mg with atropine 0.25 mg were given.

**Outcomes:**

Case 1 recovered uneventfully and was discharged on postoperative day (POD) 6. Case 2 required intensive care unit admission, re-intubation on POD 1, and re-extubation on POD 3; she was discharged on POD 23 without new neurologic deficits.

**Lessons:**

In ALS, recurarization can occur despite seemingly adequate sugammadex reversal. When rocuronium is used, sugammadex is recommended for reversal, with vigilant quantitative neuromuscular monitoring and extended post-extubation observation to detect delayed weakness.

## 1. Introduction

Amyotrophic lateral sclerosis (ALS) is a rare disease that affects motor neurons. It is characterized by the degeneration of both upper and lower motor neurons.^[1]^ Clinical features including dysphagia, dysarthria, sialorrhoea, spasticity, weakness, muscular atrophy, and respiratory insufficiency may be present.^[1]^ Approximately 50% of patients with ALS die within 3 years of onset, which is a common cause of death from respiratory failure.^[2]^

Patients with ALS may require general anesthesia for various surgical procedures.^[2]^ Depolarizing neuromuscular blocking agents, such as succinylcholine, should be contraindicated in patients with motor neuron disease because of the potential for fatal hyperkalemia and cardiac arrest.^[3]^ Nondepolarizing neuromuscular blocking agents can be used in these patients, but due to higher sensitivity, lower doses should be administered.^[2,3]^

Sugammadex is a modified γ-cyclodextrin used to reverse the effects of aminosteroid-structured nondepolarizing neuromuscular blocking agents such as rocuronium.^[4]^ Sugammadex works by encapsulating rocuronium in a 1:1 ratio and forming a very tight complex, which is excreted in the urine.^[4]^ Sugammadex can rapidly reverse any depth of rocuronium-induced muscle relaxation, and its effect is dose-dependent.^[5,6]^ Successful reversal of rocuronium-induced neuromuscular blockade with sugammadex has been reported in patients with ALS.^[7–9]^ However, care should be taken when managing these patients. Herein we report 2 cases of ALS patients, one of them developed recurarization after sugammadex reversal.

## 2. Case presentation

### 2.1. Case 1

A 67-year-old woman (weight, 45 kg; height, 160 cm) was diagnosed with ALS 3 years ago and was scheduled for percutaneous endoscopic gastrostomy (PEG) due to dysphagia. Other features included dyspnea, dysarthria, and spontaneous clonus. She was diagnosed with coronary heart disease 2 years ago, treated with rosuvastatin, and placed on noninvasive positive-pressure ventilation for 1 year. Her muscle strength was grade 2 and 3 in the right and left extremities, respectively. Renal function (plasma creatinine 49 µmol/L) and liver function (alanine transaminase 15 IU/L, aspartate transaminase 27 IU/L, total bilirubin 17.7µmol/L) were normal. She refused sedation and regional anesthesia during the procedure which was a common practice.^[10]^ We therefore decided to perform general anesthesia after discussion with both patient and endoscopist.

In the operating room, the patient was routinely monitored with electrocardiogram, noninvasive blood pressure, pulse oxygen saturation, and bispectral index. After preoxygenation with 100% oxygen, anesthesia was induced using 50 mg propofol(Propofol Injection, Fresenius Kabi Deutschland GmbH), 3 mg etomidate(Etomidate Injectable Emulsion, Jiangsu Nhwa Pharmaceutical Co., Ltd.), and 8 µg sufentanil(Sufentanil Citrate Injection, Yichang Humanwell Pharmaceutical Co., Ltd.). The train-of-four (TOF) count was 4 after loss of consciousness. We then administered 1 dose of 10 mg (0.2 mg/kg) rocuronium(Esmeron, N.V. Organon). Two minutes later, the TOF count reached 0 and the patient was intubated. Anesthesia was maintained with infusion of propofol (100 mg/hour) and remifentanil(Remifentanil Hydrochloride for Injection, Yichang Humanwell Pharmaceutical Co., Ltd.) (effect-site concentration 2.0 ng/mL). The operation lasted 12 minutes. The TOF count remained 0 at the end of surgery. We administered 90 mg (2 mg/kg) sugammadex(Bridion, Patheon Manufacturing Services LLC) when the bispectral index was more than 80. After 1 minute, the TOF count reached 4. After an additional 2 minutes, the TOF ratio reached 98% and the patient was conscious, oriented, and cooperative and breathed spontaneously. She was extubated, put on noninvasive positive-pressure ventilation again, and monitored in the postanesthesia care unit with stable vital signs. The patient was transferred to the general ward 30 minutes later, and was discharged to home on postoperative day 6 without any complications.

### 2.2. Case 2

A 68-year-old woman (weight, 53 kg; height, 158 cm) was diagnosed with ALS 2 years ago and was scheduled for PEG due to dysphagia. Her other clinical manifestations included dysarthria, dyskinesia, and sialorrhea. Physical examination showed difficulty in closing eyes and bulging cheeks, tongue muscle atrophy and fibrillation, upper-limb muscle strength grade 4, lower-limb muscle strength grade 5, symmetric limb muscle tension, symmetric upper-limb reflexes, and hyperactive lower-limb reflexes. Bilateral Babinski signs were negative. She was diagnosed with hypertension and type 2 diabetes mellitus 10 years ago and treated with fosinopril, amlodipine, and metformin. She underwent tonsillectomy 40 years ago and left adrenal tumor resection 8 years ago. Her renal function (plasma creatinine 51 µmol/L) and liver dysfunction (alanine transaminase 29 IU/L, aspartate transaminase 26 IU/L, total bilirubin 8.3 µmol/L) were normal. Pulmonary function test was not performed. General anesthesia was planned after discussion with patient and endoscopist.

Monitoring in the operating room was same as above. After preoxygenation, anesthesia was induced with 40 mg propofol, 8 mg etomidate, and 10 µg sufentanil. The TOF count reached 0 after administering 1 dose of 30 mg (0.6 mg/kg) rocuronium. Endotracheal intubation was performed. Anesthesia was maintained with 150 mg/hour propofol and target-controlled infusion of remifentanil at an effect-site concentration of 2.0 ng/mL. The operation lasted for 15 minutes. At the end of surgery, 200 mg sugammadex (3.8 mg/kg) was administered when the bispectral index was recovered to more than 75 and the TOF count reached 1. Four minutes later, the TOF ratio recovered to 92%; the patient was able to open eyes and follow commands and breathed spontaneously with a tidal volume of more than 300 ml. She was extubated and was transferred to the postanesthesia care unit.

Three minutes after extubation, the patient complained of dyspnea in the postanesthesia care unit. Monitoring showed progressive oxygen desaturation, sinus tachycardia, and hypertension. Physical examination revealed suprasternal, supraclavicular, and intercostal inspiratory retractions. Mask-assisted ventilation was immediately performed. As recorded in the medical record, an immediate arterial blood gas analysis documented severe hypercapnia (original numeric values unavailable), whereas bedside chest ultrasound examination was unremarkable. According to the signs and symptoms, we considered the recurrence of residual neuromuscular block and administered 2 mg neostigmine(Neostigmine Methylsulfate Injection, Zhejiang Xianju Pharmaceutical Co., Ltd.) and 0.25 mg atropine(Atropine Sulfate Injection, Henan Runhong Pharmaceutical Co., Ltd.). Two minutes later, the respiratory situation gradually improved and the vital signs were stabilized. The patient was transferred to the intensive care unit. However, the patient was reintubated due to acute respiratory failure on postoperative day 1 and re-extubated on postoperative day 3. She was transferred to the general ward on postoperative day 8 and discharged to home on postoperative day 23.

## 3. Discussion

ALS is a rare disease, with an incidence of 2 to 3 cases per 100,000 people in Europe but lower in east Asia (approximately 0.8 cases per 100,000 people).^[1]^ The etiology of most ALS cases remains unclear, although a subset of patients has familial history and mutations in genes.^[1]^ In addition to the degeneration of motor neurons, non-motor neuraxis and nonneuronal cells might also be involved in the pathogenesis.^[1,11]^ There is no curative therapy for ALS, both pharmacological and nonpharmacological interventions are used to improve function and prolong survival.^[1,12]^ The course of this disease is progressive, and most patients die from respiratory failure.^[1]^

For ALS patients undergoing general anesthesia, depolarizing neuromuscular blockers such as succinylcholine are contraindicated because of the risk of lethal hyperkalemia^[3]^; nondepolarizing neuromuscular blockers should be avoided when possible because of increased sensitivity and prolonged effects.^[2,3]^ If nondepolarizing neuromuscular blocking agents have to be used, the dose should be reduced and titrated according to neuromuscular monitoring.^[13,14]^ It should be noted that, for patients with ALS, the normal twitch amplitude may be reduced, and the monitoring device should be calibrated before use.^[15]^

Sugammadex is increasingly used in patients with neuromuscular or motor neuron disease.^[13,14]^ It reverses the effect of rocuronium more rapidly without significant adverse hemodynamic effects when compared with neostigmine.^[16–18]^ The effect of sugammadex is dose-dependent; a dose of 2 mg/kg and 4 mg/kg is recommended to reverse moderate and deep muscle relaxation, respectively.^[4,19]^ There is no recommended dose of sugammadex in patients with ALS, but the above dosing regimens seem also effective. For example, in an ALS case reported by Kelsaka et al,^[8]^ 20 mg (0.28 mg/kg) rocuronium was used for anesthesia induction and an additional 10 mg for maintenance. At the end of surgery, muscle strength and tidal volume were not enough despite a TOF ratio > 90%; both of which completed recovered after 2 mg/kg sugammadex. Yoo et al^[9]^ also reported an ALS case undergoing general anesthesia with 20 mg rocuronium. The TOF count was 0 at the end of surgery, but the TOF ratio recovered to 115% within 4 minutes after administration of 200 mg sugammadex (4 mg/kg).

In case 1 of our patients, deep muscle relaxation (TOF count = 0) was completed reversed with 2 mg/kg sugammadex at the end of surgery. Whereas in case 2, neuromuscular block recurred at 7 minutes after a complete reversal of rocuronium-induced block (TOF ratio of 92%) with a higher dose (3.8 mg/kg) sugammadex; the recurred neuromuscular block was reversed with an additional 2 mg neostigmine. Similar phenomenon was also observed by others. Chun et al^[20]^ reported a 71-year-old man with ALS who received general anesthesia with rocuronium. Sugammadex at a dose of 4 mg/kg was used to reverse deep neuromuscular block. They measured a TOF count of 4 and a TOF ratio of 73% at 8 minutes after sugammadex administration. However, the TOF count then decreased to 1 to 3 and the TOF ratio decreased to 54%. Both parameters completed recovered after an additional 50 mg sugammadex. Recurarization after sugammadex also occurred in patients without ALS.^[21]^ The phenomenon usually occur after a large dose rocuronium and/or a small dose sugammadex,^[20,21]^ possibly due to redistributed of rocuronium from the peripheral compartment and insufficient sugammadex.^[4,19]^ Indeed, in case 2 of our patients, a normal induction dose of rocuronium (0.6 mg/kg) was administered despite a short duration surgery.

According to the European Federation of Neurological Societies guidelines, noninvasive positive-pressure ventilation should be considered for ALS patients when there are symptoms or signs of respiratory insufficiency.^[22]^ Careful preoperative evaluation may help to identify those with or at high risk of respiratory insufficiency.^[22–24]^ Furthermore, patients with bulbar dysfunction tend to progress rapidly to acute respiratory failure.^[23]^ In case 1 of our patients, noninvasive positive-pressure ventilation was initiated before surgery and continued after extubation; postoperative recovery was uneventful in this patient. Whereas in case 2, although noninvasive positive-pressure ventilation was not required before surgery, the patient still developed acute respiratory failure on postoperative day 1. Early noninvasive respiratory support might be helpful to prevent re-intubation in this patient.^[25]^

The primary reason for prioritizing rocuronium in this case was the availability of sugammadex, which enables rapid and predictable reversal of neuromuscular block across the full range from shallow to deep levels. This capability is particularly critical in patients with ALS, who have respiratory muscle weakness and a high risk of residual neuromuscular blockade. Recent guidelines and reviews consistently recommend sugammadex as the first-line agent for antagonizing rocuronium-induced block, with stronger evidence than neostigmine for reducing residual blockade and postoperative pulmonary complications.^[26,27]^

Cisatracurium is indeed primarily metabolized via Hofmann elimination and only weakly dependent on hepatic or renal clearance; however, at typical intubating doses it has a relatively slow onset (~188 seconds) and is therefore unsuitable for rapid sequence induction. In our PEG intubation setting, we require reliable and rapid intubating conditions with controllable reversal, which aligns better with a “rocuronium plus sugammadex” strategy. In addition, rocuronium at a typical intubating dose has a faster onset (achieving intubating conditions in ~92 seconds), making it appropriate for PEG, where the swallow reflex is absent and aspiration risk is high.^[28]^

In Case 2, we used a standard intubating dose of rocuronium (0.6 mg/kg) based on the assessment of airway and aspiration risks (PEG with potential gastric content regurgitation) and the need for rapid, stable intubating conditions at that time. We also preplanned reversal with sugammadex and used quantitative neuromuscular monitoring to guide dosing throughout. Given the heterogeneity of responses to neuromuscular blockers in ALS, and prior reports of motor neuron disease cases safely managed with standard-dose rocuronium plus sugammadex, we explicitly frame this case as an individualized trade-off rather than a general recommendation.^[29]^ In the Discussion, we also report the occurrence of recurarization after reversal in Case 2 and relate it to established mechanisms – insufficient sugammadex dosing and peripheral redistribution of rocuronium – underscoring the importance of quantitative monitoring and adequate dosing.^[30]^

Given the clinical context and availability of agents at that time, we selected neostigmine for reversal in Case 2 and did not administer additional sugammadex; this may have introduced bias associated with the choice of reversal strategy and constitutes a limitation of the present case report. Most guidelines and reviews favor a “single-agent first” strategy: for reversal of rocuronium-induced neuromuscular block from deep to shallow levels, adequately dosed sugammadex is the first choice; only in very shallow block may neostigmine be used as an alternative. Routine “combination (dose-reduction)” regimens are not recommended, to avoid increased cholinergic adverse effects and added dosing complexity.^[27]^

For Case 2, the original numeric values from the initial arterial blood gas report are no longer retrievable because the paper printout was not preserved. We conducted a targeted search through the medical records archive, the laboratory information system, and the analyzer/device logs, but no results were found. To avoid unwarranted inference, we therefore report only a qualitative characterization of hypercapnia, supported by the patient’s clinical findings. This missing primary data may reduce the verifiability of our observations and constitutes a major limitation of this report.

In summary, sugammadex should be used in ALS patients after general anesthesia with rocuronium. Careful monitoring must be performed for possible recurarization even after sugammadex reversal. Early noninvasive respiratory support may be helpful in these patients.

## Author contributions

**Investigation:** Ying Zhang.

**Resources:** Xiao Hu, Xue Li, Lin Han, Ting Ding, Dong-Xin Wang.

**Supervision:** Xiao Hu, Xue Li, Lin Han, Ting Ding, Dong-Xin Wang.

**Writing – original draft:** Yun-Wen Wang, Ying Zhang, Ting Ding, Dong-Xin Wang.

**Writing – review & editing:** Yun-Wen Wang, Ying Zhang, Ting Ding, Dong-Xin Wang.
